# From classical practices to precision agriculture: a multidisciplinary review of tea (*Camellia sinensis*)

**DOI:** 10.3389/fpls.2026.1848455

**Published:** 2026-07-06

**Authors:** Muhammet Yildiz, Mehmet Ali Mert, Sheikh Mansoor

**Affiliations:** 1 Department of History, Faculty of Arts and Sciences, Recep Tayyip Erdogan University, Rize, Türkiye; 2 Department of International Relations, Political History, Faculty of Economics and Administrative Sciences, Recep Tayyip Erdoğan University, Rize, Türkiye; 3 Department of Horticulture, Faculty of Agriculture, Recep Tayyip Erdoğan University, Pazzar, Rize, Türkiye

**Keywords:** *Camellia sinensis*, climate change, global trade, machine learning, organic farming, precision agriculture, remote sensing

## Abstract

Tea (*Camellia sinensis*) is one of the most widely consumed beverages worldwide and a crop of enduring cultural, economic, and scientific significance. Although many studies has examined cultivation and trade, no previous review has integrated tea’s long-term domestication history with recent advances in precision agriculture and digital technologies. This review addresses that gap by providing a multidisciplinary synthesis that links the historical, biological, agroecological, and technological dimensions of tea production. This review demonstrates that tea productivity and quality are strongly influenced by interactions among genotype, environment, and management practices, while climate variability increasingly disrupts these relationships. At the same time, precision agriculture technologies offer substantial potential to improve disease detection, optimise resource use, and support data-driven decision-making. However, their adoption remains uneven because of high implementation costs, limited accessibility for smallholder farming systems, and challenges related to data infrastructure. Based on this synthesis, we propose that future research and policy should prioritise: (i) the region-specific integration of digital technologies with traditional cultivation practices; (ii) the development of low-cost, scalable technologies suitable for smallholders; and (iii) climate-resilient cultivation strategies adapted to shifting agroecological conditions. These targeted interventions are essential for improving productivity, sustainability, and resilience across the global tea industry.

## 1 Introduction

Tea (*Camellia sinensis)*, is one of the most widely consumed beverages globally, second only to water, and holds profound cultural, economic, and scientific significance across civilizations (Liu, 2023). Historical records and archaeological evidence indicate that tea consumption began in ancient China, before spreading across Asia, Europe, and eventually the rest of the world through trade and cultural exchange (Pan et al., 2022; Lan et al., 2023). Over time, tea became deeply embedded in global trade networks, playing a central role in economic systems and colonial expansion. Today, tea is cultivated in more than 35 countries, with major producers including China, India, Kenya, and Sri Lanka, contributing significantly to global agricultural economies and international trade (Wang, 2022; Kumarihami and Song, 2018; Suroso et al., 2025; Chang, 2015; Sarkar et al., 2025).

Historically, tea harvesting relied heavily on manual labour, emphasizing selective plucking techniques such as “one bud and two leaves,” which are critical for producing high-quality tea (Zhang et al., 2023b; Abhiram, 2024). Classical tea practices, including traditional cultivation, manual plucking, and artisanal processing techniques, have long been essential in maintaining tea quality and cultural authenticity (Sandika, 2018; Seyıs et al., 2019; Lan et al., 2023). However, the tea industry has undergone a significant transformation in recent decades due to technological advancements and evolving global challenges (Dechassa and Merga, 2022). The integration of modern technologies, such as machine vision, AI, and automated harvesting systems, has revolutionised tea production by improving efficiency, reducing labour dependency, and enabling precision agriculture (Yang et al., 2023; Ahale et al., 2024; Kalita et al., 2025; Dechassa and Merga, 2022). Organic tea cultivation, for instance, has gained attention as a strategy to enhance environmental sustainability, improve soil health, and meet consumer demand for eco-friendly products (Li et al., 2024a, b).

This comprehensive review examines tea through an interdisciplinary perspective, encompassing its historical origins, global trade dynamics, traditional cultivation practices, technological innovations, and integrated management strategies. Rather than following a systematic review framework, the review synthesizes and contextualises knowledge from diverse scientific, agricultural, economic, and technological domains to provide a broad understanding of the factors influencing the evolution and future trajectory of the tea industry. By integrating insights across disciplines, this review aims to support the sustainable development, resilience, and modernization of global tea production systems.

## 2 History of tea cultivation

Tea is widely recognised as originating in southwest China. Traditional Chinese accounts attribute the discovery of tea to the legendary emperor Shen Nong, with descriptions of tea infusion appearing in early historical texts (Pan et al., 2022). Chemical analyses of charred residues recovered from Warring States period tombs are consistent with modern tea compounds, while plant remains identified at Han Dynasty sites demonstrate early cultivation and use (Benn, 2015). Phylogenetic analyses identify two principal domestication events in China and an additional event in India. The Chinese lineage is estimated to have diverged and later contributed to the emergence of Assam-type tea varieties in Yunnan and the Indian subcontinent (Cheng et al., 2025; Balasaravanan et al., 2003; Li et al., 2024a). The Yunnan–Guizhou–Sichuan region of southwest China exhibits the highest diversity of wild tea populations, supporting its designation as the primary centre of origin (Hasimoto, 2001). Linguistic evidence reflects historical diffusion patterns, with the term “cha” and “te” disseminated via major trade routes, including the Silk Road, the Tea Horse Road, and maritime networks, facilitating the spread of tea to Persia, Europe, and other regions of Asia (Li, 2019; Wang, 2022).

From the Tang Dynasty onward, tea culture became increasingly systematised and influential. This development is exemplified by *The Classic of Tea*, authored by Lu Yu, which codified practices related to tea cultivation, processing, and consumption (Lu, c. 760/1975). By the 19th century, black tea produced in the Wuyi Mountains had entered global markets, reinforcing China’s role as the principal geographic and cultural origin of tea cultivation (Zhang & Zhang, 2026; Grigg, 2002). From this core region, the phonetic forms “cha” (Mandarin Chinese) and “te” (Min Nan/Fujian dialect) diffused along two principal transmission routes. Overland, trade networks such as the Silk Road and the Tea Horse Road facilitated the movement of compressed tea and the “cha” term westward through Tibet, Central Asia, and the Middle East (Pan et al., 2022; Lan et al., 2023). In contrast, maritime trade routes enabled Min Nan merchants to disseminate the “te” form from Fujian to Southeast Asia, where it was subsequently incorporated into Malay and Indonesian languages and later adopted into various European languages (Li, 2019; Lu, c. 760/1975).

European engagement with tea began in the early 17th century, when the Dutch East India Company imported Chinese tea to the Netherlands in 1610, followed by adoption in England by 1658 (Wang, 2022; Balasaravanan et al., 2003; Li et al., 2024b). The growing British dependence on Chinese tea created a significant trade imbalance, prompting the East India Company to establish tea plantations in India in the mid-19th century and, by 1875, in Ceylon (modern Sri Lanka) (Zhang and Zhang, 2026; Wang, 2022). By the late 19th century, tea cultivation had expanded well beyond its original centre of domestication, reaching regions in Africa (including Kenya and Tanzania), South America (notably Brazil and Argentina), and parts of the South Pacific. This expansion marked tea’s transformation from a regionally confined Chinese crop into a globally significant agricultural commodity (Balasaravanan et al., 2003; Li et al., 2024b; Hasimoto, 2001).

## 3 Most common types of tea and processing

### 3.1 Green tea

Green tea, a non-oxidized form of tea derived from tea, represents the earliest and most traditional type of tea developed in China (Lan et al., 2023). Its origin is closely linked to the initial domestication and use of tea plants in southwest China, where both archaeological and historical evidence indicate that tea was first consumed as a medicinal infusion before evolving into a daily beverage (Lan et al., 2023; Cheng et al., 2025). Unlike black tea, green tea undergoes minimal processing, with freshly harvested leaves subjected to heat treatment either by steaming or pan-firing to inactivate oxidative enzymes, thereby preserving the natural green colour, polyphenols, and characteristic fresh flavour (Kumar et al., 2018; Xu and Chen, 2002).

The historical development of green tea is deeply embedded in Chinese culture. By the Tang Dynasty, tea drinking had become widespread, and methods of cultivation, preparation, and consumption were systematically documented in *The Classic of Tea* by Lu Yu, which emphasised techniques for producing high-quality tea (Lan et al., 2023). During this period and into the Song Dynasty, tea preparation evolved from compressed tea cakes to loose-leaf forms, and green tea became the dominant type consumed. Famous varieties such as Longjing (Dragon Well), Biluochun, and Huangshan Maofeng later emerged as representative high-quality green teas, particularly valued for their aroma, appearance, and taste (Lan et al., 2023).

The spread of green tea beyond China occurred through both cultural exchange and trade. It was introduced to Japan as early as the 8th century by Buddhist monks, where it became integral to Japanese culture, eventually giving rise to the tea ceremony and the production of powdered green tea (matcha). In contrast, while green tea was initially exported to Europe in the early phases of the global tea trade, it was later overshadowed by black tea due to the latter’s longer shelf life and suitability for long-distance transport. Nevertheless, green tea has remained the predominant form of tea consumption in East Asia, particularly in China, Japan, and Korea.

In modern times, green tea has gained renewed global attention due to its perceived health benefits, largely attributed to its high content of catechins and other antioxidant compounds (Cabrera et al., 2006). Scientific studies have associated green tea consumption with various health-promoting effects, including anti-inflammatory, cardiovascular-protective, and metabolic benefits (Kochman et al., 2020; Wang et al., 2021; Lan et al., 2023; Pastore and Fratellone, 2006).

### 3.2 Black tea

Black tea, a fully oxidized product of tea, originated in southwest China, which is widely recognised as the primary centre of tea evolution, domestication, and early utilization (Zhang et al., 2023b; Lan et al., 2023; Cheng et al., 2025). The development of black tea is particularly associated with the Wuyi Mountains in Fujian Province, where innovations in processing, specifically withering, rolling, enzymatic oxidation, and drying, led to the production of the earliest forms of fully oxidized tea. Among these, Zhengshan Xiaozhong (Lapsang Souchong) is widely regarded as the first black tea produced for export (Liu, 2023; Pan et al., 2022; Lan et al., 2023). The emergence of black tea occurred later, during the Ming and Qing periods, when increased domestic consumption and expanding international trade created demand for teas with longer shelf life and greater transport stability (Liu, 2023; Pan et al., 2022). These conditions favoured the development of fully oxidized teas, which were more suitable for long-distance export (Benn, 2015; Lan et al., 2023).

The global spread of black tea was closely linked to early modern trade networks and European colonial expansion. Tea was first introduced to Europe in the early 17th century, where it rapidly became a popular beverage, particularly in Britain (Saberi, 2010; Mackillop, 2015; Ellis et al., 2015, Ellis et al., 2024). As demand grew, reliance on Chinese tea created significant trade imbalances, prompting colonial powers to establish independent sources of production. In the 19th century, the British successfully introduced tea cultivation to India, particularly in Assam and Darjeeling, using both indigenous tea varieties (*C. sinensis* var. *assamica*) and imported Chinese germplasm (Saberi, 2010; Mackillop, 2015; Ellis et al., 2015, Ellis et al., 2024). Tea cultivation was subsequently expanded to Sri Lanka (formerly Ceylon) following the collapse of coffee plantations due to disease, and to Southeast Asia, notably Java, under Dutch colonial administration. Later, tea production spread to Africa, including Kenya and Tanzania, and to parts of South America and the Pacific, completing its transformation into a global agricultural commodity (Grigg, 2002; Wenzlhuemer, 2008; Wickramasinghe and Cameron, 2004; Wijetunga and Sung, 2015; Sandika, 2018).

Today, black tea dominates global tea consumption, particularly in Western countries, and represents a major component of the international tea trade. The history of black tea illustrates a progression from localised innovation in China to widespread global cultivation, driven by technological development, trade expansion, and colonial economic strategies (Sharangi et al., 2014; Pan et al., 2022; Yong-Mei et al., 2022). From the 16th century onward, tea spread westward through trade networks into Europe and subsequently to other regions, including the Ottoman Empire (Yildirim and Karaca, 2022; Yildirim and Bohne, 2024). Although Turks were introduced to tea earlier in Central Asia before migrating into Anatolia in the 11th century, widespread consumption and systematic cultivation in Türkiye did not occur until much later (Seyıs et al., 2019; Yildirim and Karaca, 2022). Initially, coffee dominated Ottoman beverage culture, and tea remained a relatively minor drink until the late 19th and early 20th centuries (Yildirim and Bohne, 2024).

The Black Sea coastal region, especially around Rize, was identified as suitable for tea cultivation due to its humid climate, high rainfall, and acidic soils, conditions like those found in traditional tea-growing regions. As a result, tea plantations were established, and cultivation expanded rapidly across provinces such as Rize, Trabzon, Artvin, and Giresun (Ercisli, 2012; Yildirim and Karaca, 2022). By the late 20th century, Türkiye had become one of the leading tea-producing countries, ranking among the top producers globally and supporting a significant agricultural sector (Güneroğlu et al., 2018; Yildirim, 2022). Today, Turkish tea production is concentrated almost entirely in the eastern Black Sea region, where climatic suitability allows for high yields and consistent production. In addition to its agricultural importance, tea has become deeply embedded in Turkish culture and daily life (Güneroğlu et al., 2018; Yildirim, 2022). It is consumed throughout the day, served in characteristic tulip-shaped glasses, and plays a central role in social interaction and hospitality (Ercisli, 2012; Yildirim and Karaca, 2022).

## 4 Tea biology and agronomy

**Figure 1:**
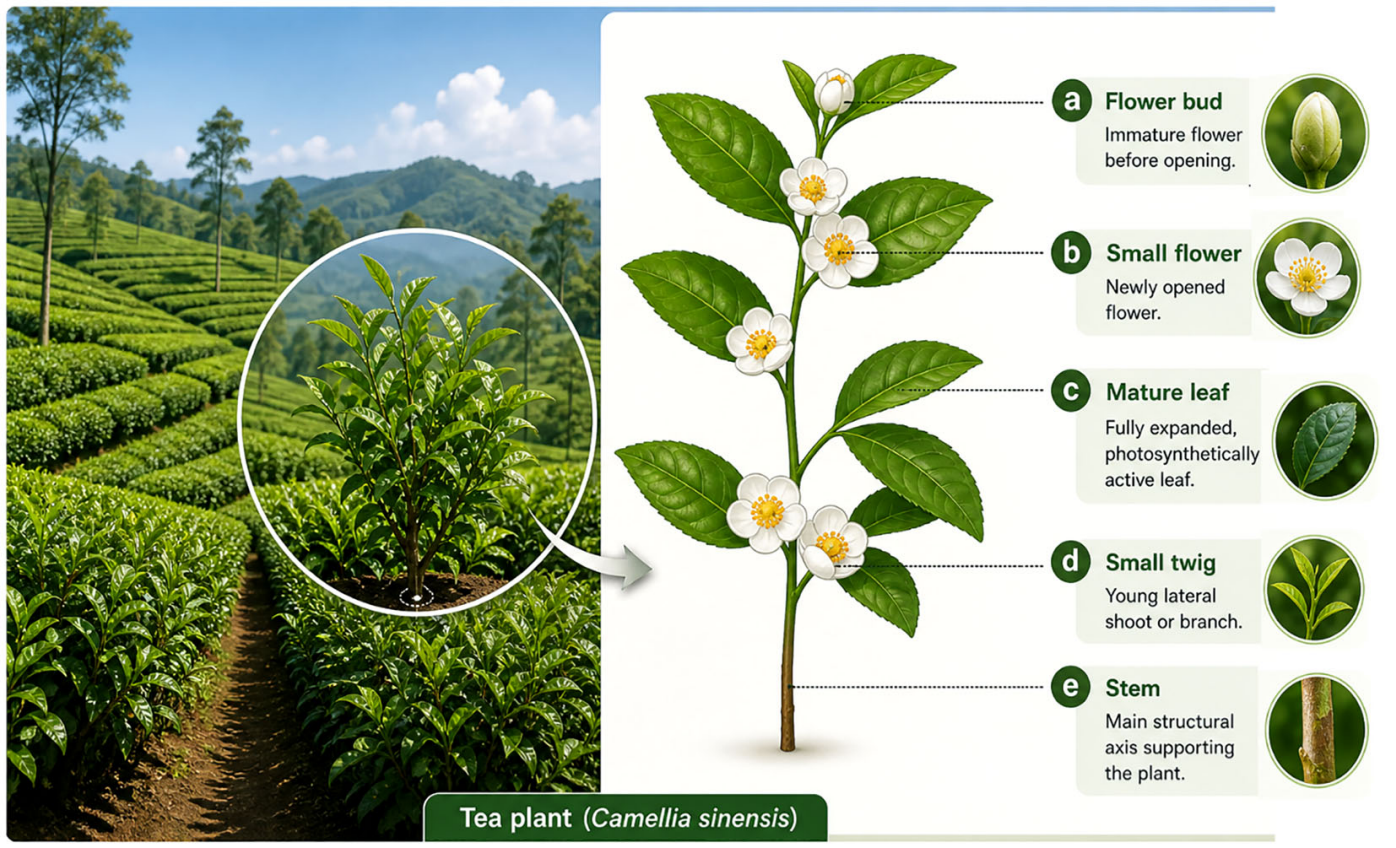
Morphological features of the tea and field context. The right panel presents a schematic representation of key morphological components, including **(A)** flower bud (immature stage), **(B)** small flower (recently opened), **(C)** mature leaf (fully expanded and photosynthetically active), **(D)** small twig (young lateral shoot), and **(E)** stem (main structural axis).

Tea plant biology and agronomy form the foundation of successful tea cultivation, as the growth, yield, and quality of tea are closely linked to the botanical characteristics of the plant and the environmental conditions under which it is grown (Hajiboland, 2017; Owuor et al., 2011; Wang et al., 2024). Tea is derived from the evergreen shrub tea, which belongs to the family Theaceae. It is a perennial plant characterised by glossy, dark-green leaves, small, white, fragrant flowers, and a deep root system that allows it to survive in diverse conditions (De Costa et al., 2007; Wachira et al., 2013). The plant is typically maintained as a low bush (about 1–1.5 meters in height) through regular pruning to facilitate harvesting. The economically important part of the plant is the young, tender shoots consisting of a bud and the top two leaves, as shown in **Figure 1**, which are rich in chemical compounds such as catechins, caffeine, and amino acids that determine tea quality (Purohit, 2008; Ariyarathna et al., 2011; Mahmood et al., 2010). The plant exhibits continuous vegetative growth under suitable climatic conditions, enabling multiple harvests throughout the year.

The cultivation of tea is strongly influenced by a narrow range of environmental conditions, which explains its restriction to specific agroecological zones. Tea plants are best suited to humid tropical and subtropical regions where atmospheric moisture remains relatively high throughout the year. Consistent and well-distributed annual rainfall, typically between 1200 and 2500 mm, is essential to sustain continuous vegetative growth and leaf production (Hajiboland, 2017; Jayasinghe et al., 2020). Prolonged dry periods can significantly reduce yield unless irrigation is provided. Temperature is another critical determinant of tea growth and productivity (**Table 1**). Optimal development occurs within a moderate thermal range of approximately 18 °C to 30 °C (Babu et al., 2022; Sahu et al., 2024). Temperatures below this range can slow physiological processes and induce dormancy, while excessive heat can cause leaf scorch, reduce photosynthetic efficiency, and ultimately diminish both yield and quality (Oluoch et al., 2018; Jayasinghe et al., 2020; Babu et al., 2022). Similarly, frost events are particularly damaging, as they can injure tender shoots and impair regrowth (Wang et al., 2024; Que and Zhao, 2024).

**Table 1:** Optimal environmental conditions and their roles in tea growth and quality.

Factor	Optimal range	Importance	References
Temperature	18–30 °C	Supports growth and leaf development	Tan et al., 2023
Rainfall	1200–2500 mm/year	Shoot growth	Krishnakumar et al., 2024; Malyukova et al., 2023.
Soil pH	4.5–5.5 (acidic)	Essential for nutrient absorption	Jia et al., 2024; Li et al., 2023
Altitude	0–2200 m	Quality improves with altitude	Chen et al., 2022; Ran et al., 2023
Soil type	Well-drained, loamy	Prevents waterlogging and root damage	Rahaman and Aruchamy, 2022; Layomi Jayasinghe et al., 2019
Humidity	High (70–90%)	Promotes leaf growth	Xiang et al., 2025; Ran et al., 2023; Han et al., 2018a.

Soil characteristics play a fundamental role in successful tea cultivation. The crop requires well-drained soils to prevent waterlogging, which can damage the root system and promote disease. Tea plants are highly adapted to acidic conditions, with an optimal soil pH between 4.5 and 5.5; alkaline soils inhibit nutrient availability and are generally unsuitable for cultivation (Zhang, 2018; Yan et al., 2018; Huu Chien et al., 2019). In addition, deep soils enriched with organic matter are preferred, as they enhance water retention, nutrient supply, and root development, all of which contribute to sustained plant vigour (Yokota et al., 2005; Shao et al., 2024). Altitude further influences both the growth dynamics and the biochemical composition of tea leaves (Khormali et al., 2007; Muzib et al., 2023). At higher altitudes, typically above 1000 meters, cooler temperatures slow leaf growth, allowing for greater accumulation of secondary metabolites such as polyphenols and aromatic compounds. This results in teas with more complex flavour profiles and enhanced aroma (Han et al., 2017). Conversely, tea grown in lowland areas tends to grow more rapidly, leading to higher yields but often producing leaves with less concentrated flavour and comparatively lower sensory quality (Özdemir et al., 2018; Pulungan et al., 2021; Ren et al., 2025).

Tea plants vary by type and region, with the most common being the China type and locally adapted varieties such as those grown in Türkiye. The China type has small leaves, grows slowly, and tolerates cold climates, making it ideal for high-altitude areas and high-quality green teas. In contrast, Turkish tea plants are adapted to the humid Black Sea environment, are more robust, and are mainly used for black tea production. Tea growth occurs in seasonal cycles called “flushes,” Periods of new leaf growth when the tea plant produces fresh shoots (typically the “two leaves and a bud” used for harvesting). These affect both yield and quality. The first flush in early spring produces the finest, most delicate leaves. The second flush yields larger, stronger-flavoured leaves with higher output. Later flushes may occur, but heavy rainfall can reduce quality. These cycles depend on climate and farming practices, making their management essential for achieving optimal yield and maintaining tea quality (Zhao et al., 2017; Owuor et al., 2008; Fernández-Cáceres et al., 2001; Peterson et al., 2004).

## 5 Agronomic practices

Agronomic practices play a pivotal role in determining the productivity, quality, and sustainability of tea cultivation systems. Key operations such as propagation, planting, pruning, and fertilization directly influence plant growth, canopy structure, and nutrient dynamics. Optimizing these practices is essential for achieving consistent yield while maintaining soil health and long-term plantation viability.

### 5.1 Propagation

Tea is propagated through both sexual and asexual methods, each with distinct advantages depending on production goals.

#### 5.1.1 Seed propagation (sexual)

Tea seed (TS), derived from the tea, represents the mature fruit and serves as the primary biological unit for plant propagation and multiplication. Tea plants typically require 4–6 years to reach reproductive maturity, after which they enter a flowering and fruiting phase lasting approximately six months, culminating in the production of fully developed seeds (Zhou et al., 2023). Mature tea seeds are characterised by a dark brown testa, firm structure, and well-developed cotyledons. Structurally, TS comprises an outer seed coat or shell (tea seed shell, TSS) and an inner kernel. Upon dehulling, the kernel can be mechanically processed to extract tea seed oil (TSO), while the residual material, referred to as tea seed meal (TSM), is often compressed into tea seed cake as a by-product (Zhou et al., 2023; Huang et al., 2023).

#### 5.1.2 Vegetative propagation

Vegetative (clonal) propagation is the predominant method in commercial tea cultivation, typically achieved through single-node or two-node stem cuttings obtained from elite mother plants and rooted under controlled nursery conditions (Banerjee, 1992; Borchetia et al., 2009). This approach ensures genetic uniformity, consistent yield, and superior leaf quality, as desirable traits are reliably maintained across planting material. Moreover, clonal propagation enables faster establishment and earlier economic returns compared to seed-based propagation. However, it requires well-managed nursery infrastructure and may result in relatively shallower root systems than those developed from seed-grown plants (Mondal et al., 2004; Mondal, 2014). Although tea can be propagated through seeds, this method is less preferred for commercial production due to high genetic variability arising from heterozygosity, leading to inconsistency in yield and quality. The development of homozygous diploid lines is further constrained by the rare occurrence or absence of natural haploids in tea. Conventional breeding efforts are also limited by the perennial growth habit and long gestation period of the crop, which significantly slow genetic improvement (Mondal et al., 2004). Consequently, a range of biotechnological approaches, including tissue culture and molecular breeding techniques, have been explored to overcome these limitations and accelerate genetic advancement in tea, with varying degrees of success (Mukhopadhyay et al., 2016).

#### 5.1.3 Tissue culture (micropropagation)

Tissue culture (micropropagation) in tea involves the *in vitro* regeneration of plants from explants under sterile laboratory conditions. This technique enables the rapid multiplication of elite genotypes and the production of disease-free planting material, making it valuable for germplasm conservation and large-scale dissemination of high-performing clones. Since the pioneering establishment of tea cell cultures by Forrest (1969) and subsequent systematic work on micropropagation by Kato (1985), extensive research has been conducted on *in vitro* shoot proliferation from apical and axillary meristems, as comprehensively reviewed by Mukhopadhyay et al. (2016). A critical requirement for successful micropropagation is the mass production of phenotypically uniform and genetically stable plantlets, ensuring that the desirable traits of superior clones are retained. However, despite its advantages, tissue culture remains cost-intensive, technically demanding, and is still limited in widespread commercial application across many tea-growing regions (Borchetia et al., 2009; Ranatunga, 2020). Moreover, instances of somaclonal variation have been reported in micropropagated tea plants using various analytical approaches, highlighting the need for rigorous quality control and genetic fidelity assessment during large-scale propagation (Mukhopadhyay et al., 2016).

### 5.2 Tea plantation

Tea establishment requires well-coordinated plantation practices to ensure optimal growth, yield, and leaf quality. These practices encompass site selection, land preparation, planting design, and early field management (Barua, 1989; Zhao, 2024). Tea is best suited to well-drained, acidic soils (pH 4.5–5.5) under humid subtropical to tropical conditions. Land preparation typically involves vegetation clearance, contour alignment in sloping terrains, and the construction of terraces to mitigate soil erosion, along with efficient drainage systems to prevent waterlogging (Barua, 1989; Jia et al., 2024). Planting material is predominantly derived from clonal cuttings raised in nurseries, ensuring genetic uniformity, high productivity, and consistent quality, although seed-derived plants may be used in breeding programs or as rootstock. Healthy nursery plants, usually 6–12 months old, are transplanted to the field (Mondal et al., 2004; Mondal, 2014). Planting systems are adapted to topography, with contour planting widely practiced in hilly regions to reduce runoff and soil loss, while square, rectangular, or hedge-row systems are used in flatter landscapes to facilitate management and harvesting operations. Typical spacing ranges from 1.0–1.5 m between rows and 0.6–1.0 m between plants, depending on cultivar and management intensity (Barua, 1989). Shade management is another critical component, where trees such as Albizia and Grevillea are integrated into plantations to regulate microclimatic conditions, reduce heat stress, and enhance soil fertility. This practice contributes to improved leaf quality, sustained productivity, and extended plantation longevity (Mondal et al., 2004).

### 5.3 Tea pruning

Pruning is a fundamental agronomic practice in tea cultivation, playing a critical role in regulating plant architecture, enhancing branching, and promoting the production of tender shoots essential for harvest (Sun et al., 2018; Zhang et al., 2021a). In the absence of pruning, tea plants exhibit continuous vertical growth, typically increasing in height by 15–20 cm annually, which reduces productivity by making plucking difficult and limiting the formation of productive shoots (Ravichandran, 2004). Regular pruning maintains bushes at a manageable height and stimulates lateral branching, thereby increasing the number of harvestable leaves (Ravichandran and Parthiban, 1998a; Ravichandran, 2004).

The biological and physiological responses to pruning are influenced by both pruning intensity and timing. Pruning severity has been shown to significantly affect shoot regeneration, yield, and quality of subsequent flushes, particularly spring tea (Sun et al., 2018). Similarly, pruning time plays a crucial role, with studies reporting maximum yields when pruning is conducted during the dormant season, such as in December. The interaction between pruning degree and timing further modulates tea quality parameters; for instance, light pruning following summer has been associated with increased yield and polyphenol content, albeit with slight reductions in theanine and caffeine levels (Xu et al., 2014; Zhang et al., 2021a).

Unpruned tea plants tend to produce a higher proportion of dormant buds compared to actively growing shoots, underscoring the importance of pruning prior to harvest to enhance productivity. Although pruning temporarily reduces yield—typically resulting in no pluckable shoots for up to three months—this loss is compensated by accelerated shoot growth during subsequent cycles (Ravichandran and Parthiban, 1998a). However, as the next pruning cycle approaches, growth rates decline, which may influence the biochemical composition of leaves and ultimately affect tea quality (Ravichandran, 2004; Zhang et al., 2021a). In practice, tea bushes are pruned at intervals of 4–5 years in major producing countries such as India and Turkey (Ravichandran, 2004; Thomas et al., 2005). Yield dynamics within this cycle are well documented, with peak productivity and shoot density typically observed during the third to fourth year after pruning, followed by a gradual decline (Zhang et al., 2021a). These findings highlight the importance of optimizing pruning schedules to balance yield, quality, and long-term plantation sustainability.

### 5.4 Tea fertilization

Fertilizer application is a critical agronomic intervention for improving tea productivity; however, the rising demand for higher yields has led to the intensified use of chemical fertilizers in many tea-growing regions (Ji et al., 2018; Huang et al., 2022). Prolonged and excessive application of synthetic fertilizers has been associated with soil degradation, including acidification, structural decline, nutrient imbalances, and a reduction in beneficial microbial diversity (Gu et al., 2019; Lal, 2009; Li et al., 2016). In contrast, organic fertilizers provide a sustainable nutrient source, enhancing nutrient-use efficiency and supporting plant growth and soil health (Cavagnaro, 2014). Comparative studies indicate that organic and inorganic fertilization regimes exert distinct effects on soil physicochemical properties and microbial community composition (Wang et al., 2017; Huang et al., 2022).

In tea systems, the incorporation of organic amendments such as cow manure has been shown to modulate key metabolic pathways, including those associated with amino acids, sugars, and fatty acids, thereby contributing to improved tea quality (Sun et al., 2021). Consequently, integrated nutrient management strategies, which combine organic inputs with mineral fertilizers, are increasingly recommended to optimise nutrient availability, enhance economic efficiency, and ensure environmental sustainability (Huang et al., 2022). Furthermore, region-specific fertilizer formulations, developed through soil testing and field experimentation—such as balanced nutrient blends (e.g., 18-8–12 N–P_2_O_5_–K_2_O)—have been proposed to improve nutrient-use efficiency across diverse tea-growing environments (Huang et al., 2022; Ruan et al., 2020; Yi et al., 2017).

## 6 Tea production and exports

Global tea production (including black, green, and other types) has grown steadily at an average annual rate of 3.2% over the past decade, reaching approximately 6.7 million tonnes in 2022. This growth has been largely driven by China, where production increased significantly due to rising domestic demand and growing health awareness, making it the dominant producer with nearly 50% of global output. India remains the second-largest producer, contributing about 20.5%, although its growth has been relatively modest and constrained by challenges such as unfavourable weather and weak demand. Among major exporters, Kenya recorded a slight increase in production, while Sri Lanka experienced a sharp decline of 15.6% in 2022 due to policy changes, economic instability, and rising input and operational costs. Global tea production increased by 1.6% in 2022 compared to 2021, primarily driven by a rise in green tea output, which compensated for declines in black tea production. Over the past decade, green tea production has expanded at a faster annual rate (4.9%) than black tea (2.1%), reflecting growing market demand and heightened consumer awareness of tea’s health benefits (**Figure 2**). Preliminary estimates suggest a modest recovery and continued growth in global tea production in 2023 (Food and Agriculture Organization, 2024).

**bFigure 2:**
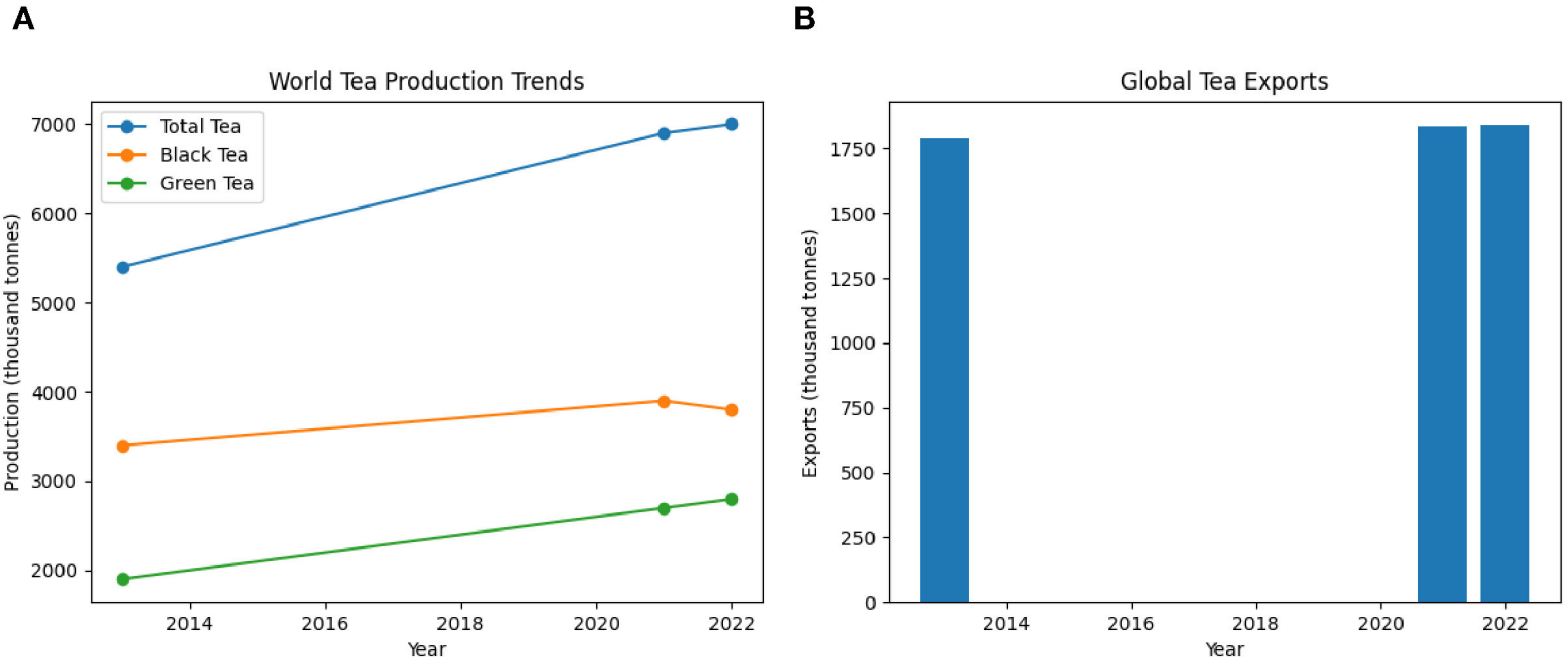
**(A)** Trends in global tea production (black, green, instant, and other types) over the last decade, showing an average annual growth rate of 3.2%. **(B)** global export (Source: FAO IGG/Tea Secretariat).

Tea remains a $17 billion-annual global industry, but production is under pressure from climate stress, labour shortages, and market imbalances. In 2023, worldwide output exceeded 6.5 Mt, yet only ≈ 26 % of that volume is exported, the rest being consumed domestically, which depresses farm-gate prices and squeezes margins (Xu et al., 2021; Kumbasaroglu, 2026). Climate change is eroding yields in key regions, models predict a 5 % drop in China, 14 % in Sri Lanka, and 25 % in Kenya by 2050. In India, the Tea Association of India warns of a 160-170 Mkg shortfall for 2024, driven by insufficient rainfall, extreme heat, and severe pest infestations; Assam’s output fell ≈ 11 % and West Bengal ≈ 21 % by July 2024 (Chen et al., 2025).

The global tea industry has experienced consistent growth due to increasing demand, population expansion, and diversification of tea products (Yang et al., 2023). Global tea production has demonstrated steady growth over the past decades. According to recent estimates, global tea production exceeds 6–7 million tonnes annually, with a consistent upward trajectory. This growth is largely driven by technological improvements, expansion of cultivation areas, and increasing global consumption. China is the largest tea producer globally, followed by India, Kenya, Sri Lanka, and Vietnam. Together, China and India account for more than half of global tea production (Bala et al., 2020).

Tea production is characterised by a strong reliance on smallholder farmers, who contribute significantly to global output. The sector provides livelihoods to millions of individuals, particularly in developing countries. Tea farming systems vary from conventional to organic practices, with increasing emphasis on sustainability (Yang et al., 2023). Recent studies indicate that technological advancements such as machine vision, AI, and automation are transforming tea production by improving efficiency and reducing labour dependency. However, labour shortages and rising production costs remain persistent challenges in the industry (Abhiram, 2024). The global tea industry holds substantial economic value, contributing billions of dollars annually to the global economy. It is a critical source of income for developing countries and supports millions of small-scale farmers. In many regions, tea farming is a primary source of livelihood, supporting rural economies and employment.

## 7 Global trade

Global tea exports grew modestly at an average annual rate of 0.5% over the past decade, reaching 1.84 million tonnes in 2022. This growth was mainly driven by green tea exports, which increased by 2.0% annually, compared to a slower 0.2% growth in black tea exports (**Figure 2B**). In 2022, black tea exports rose slightly (0.9%) due to higher shipments from Kenya and India, while exports from Sri Lanka declined sharply by 12.5% due to reduced production and economic challenges. Green tea exports increased by 1.7%, largely supported by China, which contributes over 75% of global green tea exports. Despite some recovery in Sri Lanka’s exports in 2023, global tea trade is expected to remain stagnant due to geopolitical tensions, conflicts, and global economic slowdown. These factors have reduced demand in key markets, with imports declining in Pakistan and Russia, while the United States recorded a moderate increase in tea imports in 2022 (Chang, 2015; Kumarihami and Song, 2018; Bala et al., 2020; Langford, 2021; Suroso et al., 2025). The tea trade is also shaped by regional trading blocs such as the European Union, ASEAN, and SAFTA, which influence pricing, tariffs, and market access. Export prices often differ significantly from import prices, reflecting disparities in production costs and value addition across regions (Hwang and Lim, 2017; Bala et al., 2020; Sarkar et al., 2025).

### 7.1 Global projections of tea production and exports

**Figure 3:**
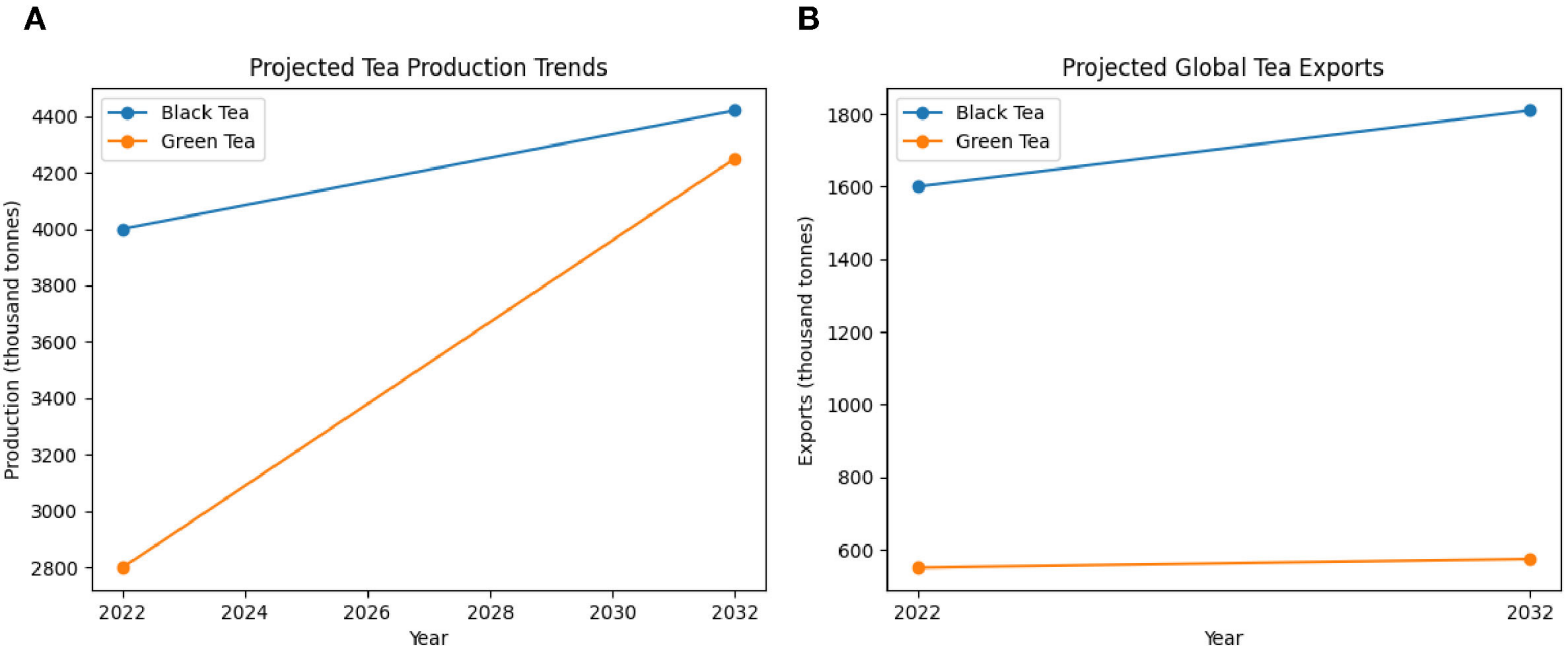
**(A)** Actual and projected production **(B)** Actual and projected exports (source: FAO IGG/Tea Secretariat).

Global black tea production is projected to grow moderately at an annual rate of 1.6%, reaching approximately 4.42 million tonnes by 2032, with recovery expected in key producing countries such as Sri Lanka. In contrast, green tea production is anticipated to expand more rapidly at 6.3% annually, reaching about 4.25 million tonnes (**Figure 3**). This growth will be primarily driven by China, where output is expected to nearly double due to improved productivity, high-yielding varieties, and better agronomic practices. Vietnam is also projected to see modest growth in green tea production, although challenges related to efficiency and quality may limit its overall gains.

Global tea exports are projected to grow steadily over the medium term, with black tea exports expected to reach 1.81 million tonnes by 2032, increasing at an annual rate of 1.2%, significantly higher than the 0.2% growth observed in the previous decade. All major exporters are anticipated to expand production, with Kenya maintaining its leading position, followed by India and Sri Lanka. India is expected to surpass Sri Lanka to become the second-largest exporter, although Sri Lanka’s exports are likely to recover at a slower pace. Green tea exports are projected to grow more rapidly, at 3.5% annually, reaching approximately 574,000 tonnes by 2032 (**Figure 3B**). This growth will be primarily driven by China and Vietnam, with China continuing to dominate the market, accounting for about 75% of global green tea exports (**Table 2**). Other key exporters, including Vietnam, Japan, and Indonesia, are also expected to experience positive growth during this period (Food and Agriculture Organization, 2024).

**Table 2:** Actual and projected production (tonnes) and growth rates (% per year).

Tea type	Region/country	Actual (2020–2022)	Projected (2032)	Growth rate (2013/2022 or 2010–2022)	Growth rate (2023–2032)
Black Tea	World	3,578,805	4,418,927	2.1	1.6
	Kenya	540,306	678,888	3.1	1.4
	India	1,346,990	1,735,310	1.4	1.7
	Sri Lanka	249,689	292,643	-2.6	0.8
	China	466,600	601,491	11.1	2.2
	Bangladesh	93,829	111,249	4.7	1.5
	Vietnam	80,000	92,028	-0.3	0.9
	Indonesia	101,767	106,864	-0.9	0.1
Green Tea	World	2,307,421	4,250,690	5.2	6.3
	China	2,060,581	3,981,697	6.1	6.8
	Japan	71,551	73,580	-1.5	0.3
	Vietnam	99,333	114,827	1.3	1.5
	Indonesia	35,000	35,352	-0.1	0.1

Values are presented in tonnes.

Growth rates represent average annual percentage changes.

Data synthesised from FAO/commodity outlook datasets (2020–2022 baseline and 2032 projections).

## 8 Organic vs conventional

Organic and conventional tea farming represent two dominant agricultural systems that differ significantly in their approaches to production, input use, environmental impact, and economic outcomes. Conventional tea farming is characterised by the use of synthetic fertilizers, pesticides, and intensive management practices aimed at maximizing yield and efficiency (Chin et al., 2010). This system has historically been the backbone of global tea production, particularly in major producing countries such as China, India, and Kenya, where high productivity is essential to meet growing global demand (Durham and Mizik, 2021). The conventional approach benefits from higher yields per unit area, relatively stable production outputs, and the ability to scale operations efficiently. Mechanization and technological innovations, including machine vision and automated harvesting systems, have further enhanced productivity and reduced labour dependency in conventional tea systems (Yang et al., 2023). However, these advantages come with environmental and social costs, including soil degradation, biodiversity loss, water contamination, and increased dependence on chemical inputs. Over time, excessive use of agrochemicals can reduce soil fertility and negatively impact long-term sustainability, raising concerns about the ecological footprint of conventional tea farming (Liu et al., 2024b).

### 8.1 Environmental impacts

In contrast, organic tea farming emphasises sustainability, environmental protection, and the use of natural inputs. Organic systems avoid synthetic fertilizers and pesticides, relying instead on compost, green manure, biological pest control, and crop diversification to maintain soil fertility and plant health (Liu et al., 2024b). This approach aligns with global trends toward sustainable agriculture and eco-friendly production practices. Organic tea farming has been shown to improve soil structure, enhance biodiversity, and reduce greenhouse gas emissions, making it a more environmentally sustainable alternative to conventional systems (Han et al., 2018b; Zhen et al., 2023; Liu et al., 2024b). Additionally, organic tea often commands higher market prices due to increasing consumer demand for chemical-free and sustainably produced products, which can enhance farm profitability despite lower yields. Studies indicate that organic farming systems may generate higher net returns in some contexts due to premium pricing and reduced input costs (Durham and Mizik, 2021). However, the transition from conventional to organic farming is not without challenges. Organic tea farming typically requires higher labour inputs, more intensive management, and greater knowledge of ecological practices. Yields in organic systems are generally lower, often by around 20–25% compared to conventional farming, which can discourage farmers from adopting organic practices, especially in regions where income stability is critical (Piyasena and Hettiarachchi, 2023; Liu et al., 2024b).

### 8.2 Socioeconomic considerations

Economic considerations play a crucial role in determining the adoption of either system. Conventional farming offers immediate economic benefits through higher productivity and lower short-term risks, making it attractive to farmers seeking stable incomes. In contrast, organic farming requires significant initial investments, including certification costs, training, and a transition period during which yields may decline before stabilizing (Liu et al., 2024b). From an environmental perspective, organic tea farming offers clear advantages. It promotes soil health through natural nutrient cycling, reduces chemical runoff, and supports biodiversity by maintaining ecological balance within tea plantations. These benefits contribute to long-term sustainability and resilience against climate change (Arhin et al., 2022; Seyıs et al., 2019; Liu et al., 2025). As climate change increasingly affects tea-growing regions, sustainable farming practices are becoming more critical to ensure long-term productivity and stability in the tea industry (Zhen et al., 2023). While conventional farming remains dominant due to its high productivity and economic reliability, organic farming is increasingly gaining traction as a sustainable and environmentally friendly alternative. As global demand for sustainable products continues to rise, and as technological advancements support more efficient production methods, the future of tea farming is likely to involve a gradual shift toward more sustainable practices that integrate the strengths of both systems.

## 9 Traditional tea farming

Traditional tea farming was a labour-intensive system that relied on manual techniques, simple tools, and indigenous knowledge passed down through generations. It began with land preparation, where farmers cleared vegetation, dug, and levelled the soil using basic hand tools such as hoes, spades, and pickaxes. In hilly regions, terraces were constructed to reduce soil erosion and improve water retention, creating a stable foundation for tea cultivation (Willson and Clifford, 2012; Kamunya et al., 2019). Planting was carried out manually using seedlings or vegetative cuttings.

Soil management and weed control were done by hand, reflecting the reliance on human labour. Farmers regularly removed weeds using small hoes and rakes to reduce competition for nutrients and water. Organic materials such as compost, animal manure, and leaf litter were applied to improve soil fertility, while mulching helped conserve moisture and regulate soil temperature. These methods promoted soil health and reduced the need for chemical inputs (Kamunya et al., 2019). Pruning was another essential activity, performed with simple tools like pruning knives and sickles. It helped maintain the shape and height of tea bushes and encouraged the growth of new shoots. Harvesting, or plucking, was the most critical stage and was done entirely by hand. Skilled workers selectively picked the top bud and two leaves, ensuring high-quality tea production (Kamunya et al., 2019). After harvesting, leaves were collected in baskets and transported manually.

**Figure 4:**
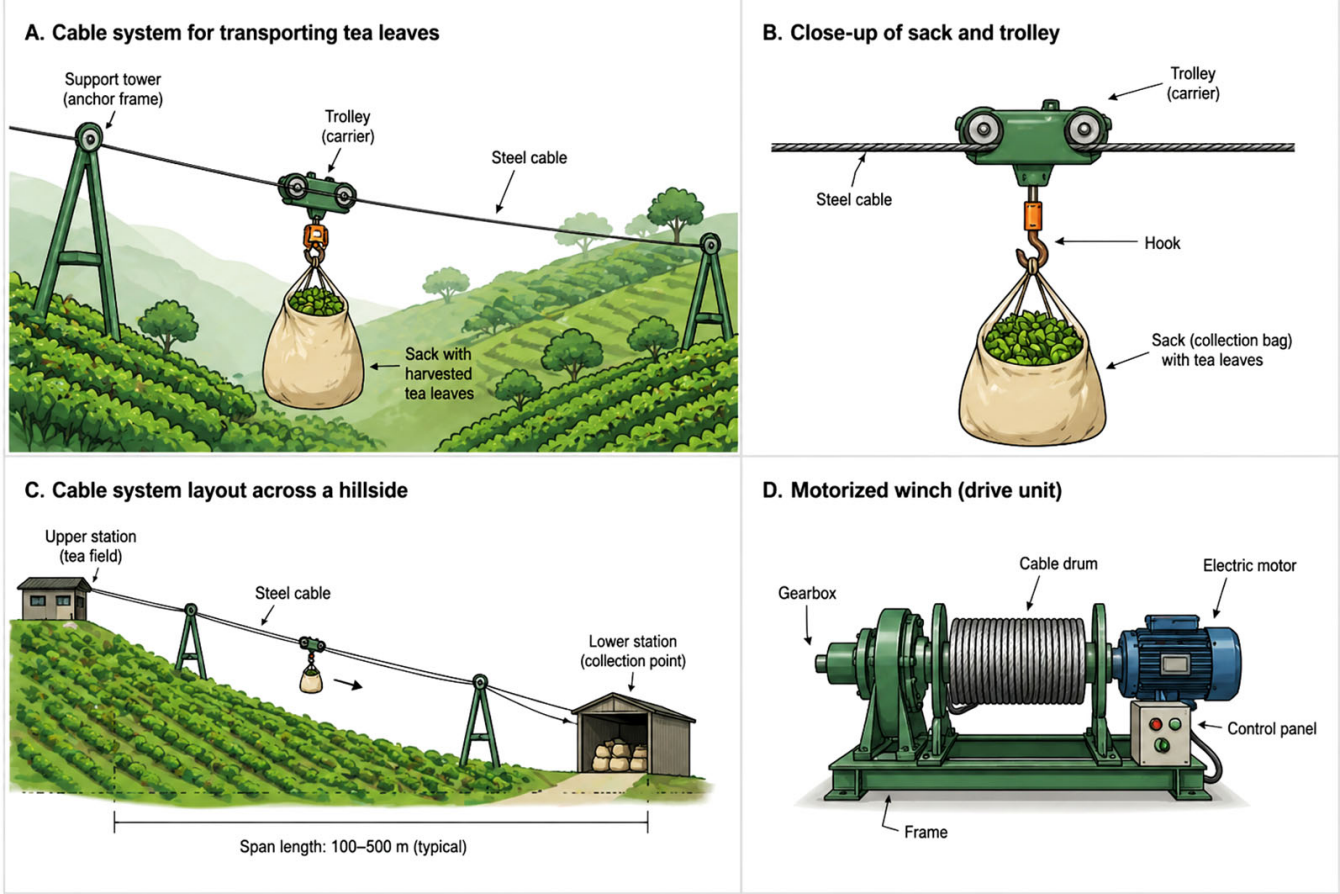
Schematic representation of a cable transport system used for moving harvested tea leaves, including **(A)** field setup, **(B)** trolley and sack mechanism, **(C)** hillside layout, and **(D)** motorised winch unit.

In tea-growing regions in Türkiye’s Black Sea region, harvesting predominantly relies on traditional and semi-mechanical methods adapted to steep terrain. Due to limited road access, farmers also use extensive improvised cable car systems consisting of steel cables, pulley mechanisms, and motorised or manual carriers to transport harvested tea across hillsides, as shown in **Figure 4**.

China, India, and Kenya each maintain distinctive tea traditions shaped by local cultivation and processing practices. In China, tea production emphasises highly regional artisanal methods such as hand-plucking, pan-firing, sun-withering, rolling, and charcoal roasting, often using carefully selected tender buds and young leaves for premium teas (UNESCO, 2026; Encyclopaedia Britannica, 2026). India traditionally follows the “two leaves and a bud” plucking standard and is known both for orthodox rolled teas and the development of the Crush–Tear–Curl (CTC) method used for strong black teas (Encyclopaedia Britannica, n.d.). Similarly, Kenya widely applies the “two leaves and a bud” harvesting practice, particularly through smallholder hand-plucking systems, while specializing in CTC black tea production and newer specialty varieties such as purple tea cultivars rich in antioxidants (Tea Directorate Kenya, 2026).

## 10 Modernization in tea agriculture

**Figure 5:**
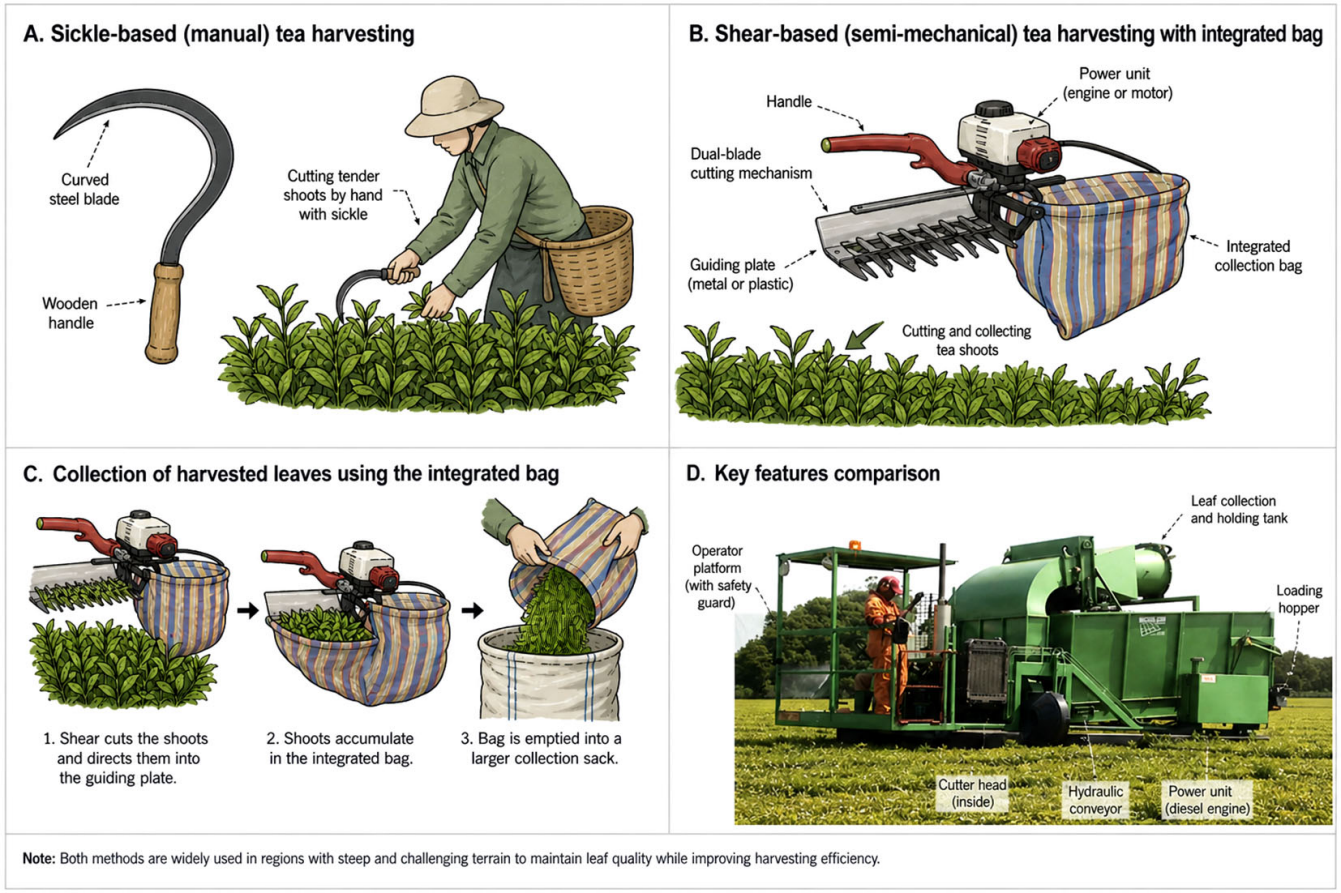
Comparison of tea harvesting methods, including **(A)** manual sickle-based harvesting, **(B)** semi-mechanical shear harvesting with integrated collection bag, **(C)** leaf collection process, and **(D)** key features of mechanised harvesting systems.

Modern tea harvesting increasingly utilizes mechanized equipment to enhance operational efficiency, harvesting accuracy, and adaptability under varying field conditions. Contemporary harvesting machines are designed to be lightweight, portable, and user-friendly, enabling effective operation across diverse terrains, including sloped and uneven landscapes. Advances in machine design have also improved leaf collection, handling, and transportation through integrated harvesting and storage systems. Furthermore, the adoption of efficient power transmission and control mechanisms has enhanced machine performance, reduced labour requirements, and increased harvesting productivity. These technological developments have significantly contributed to the modernization and sustainability of tea harvesting operations (**Figure 5**).

**Figure 6:**
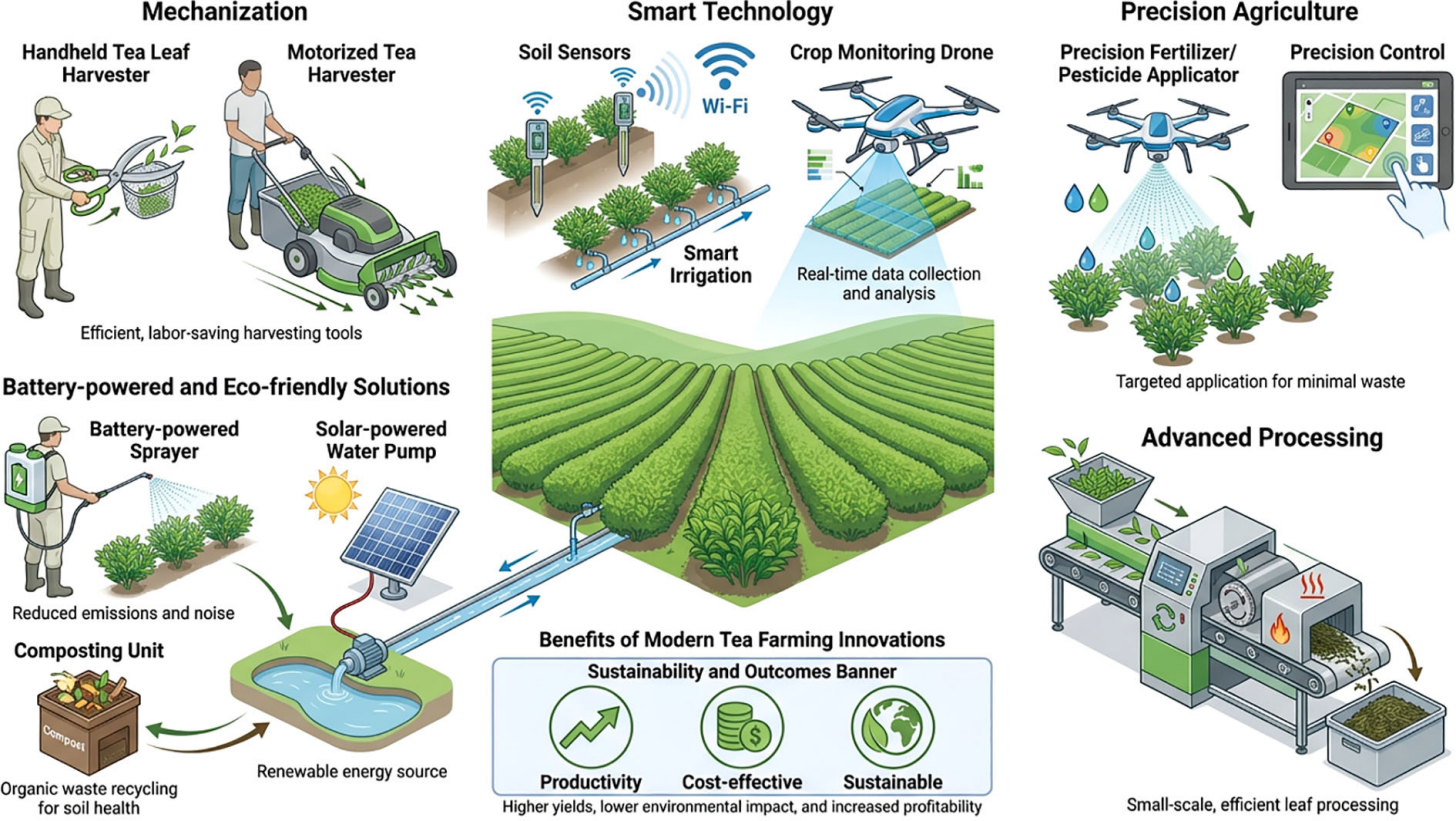
The image shows modern tea farming innovations integrating mechanization, smart technologies, and precision agriculture, including motorised harvesting, IoT-based monitoring, drone-assisted crop analysis, targeted input application, eco-friendly energy solutions, and advanced processing systems to enhance productivity, efficiency, and sustainability.

Tea farming is being transformed through the integration of mechanization, smart technologies, and precision agriculture. Traditional labour-intensive practices are increasingly replaced by handheld and motorised harvesters, improving efficiency and reducing labour dependency (Abhiram, 2024). The adoption of smart technologies, such as soil sensors, IoT-based irrigation systems, and drone-assisted crop monitoring, enables real-time data collection and informed decision-making. Precision agriculture further enhances resource efficiency through targeted application of fertilizers and pesticides, minimizing waste and environmental impact (Arhin et al., 2024). The innovations collectively enhance productivity, reduce costs, and promote environmental sustainability in modern tea farming systems (**Figure 6**).

**Figure 7:**
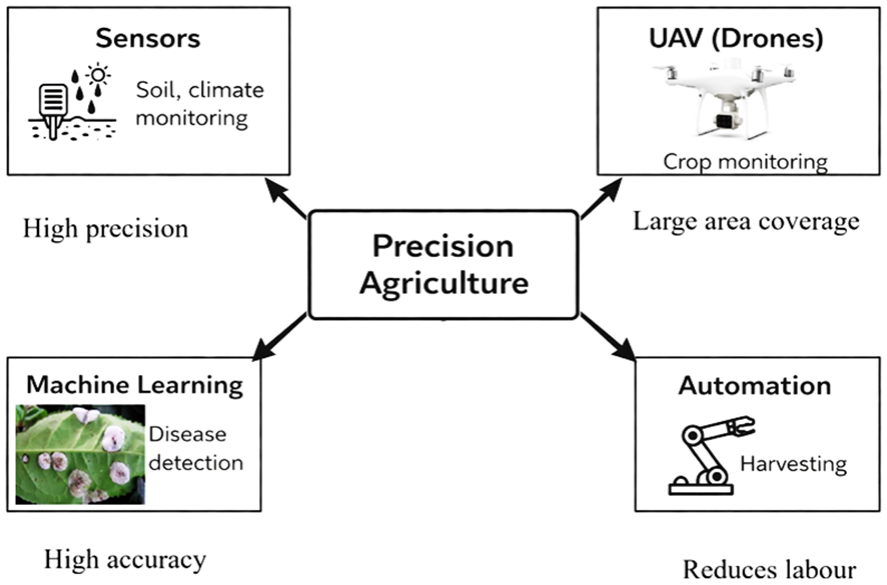
Components of precision agriculture in tea farming, illustrating the integration of sensors for soil and climate monitoring, UAV-based crop surveillance, ML for disease detection, and automation for harvesting, collectively enhancing accuracy, efficiency, and labour optimization.

Advancements in tea farming, harvesting, and monitoring technologies have significantly transformed the traditional tea industry into a more efficient, data-driven, and sustainable agricultural system. In recent years, the integration of modern technologies such as sensors, unmanned aerial vehicles (UAVs), and machine learning (ML) has enhanced productivity, reduced labour dependency, and improved tea quality (Zhang et al., 2021b; Li et al., 2022; Zhuang et al., 2025; Li et al., 2026). One of the key developments in tea farming is the use of sensor-based systems for monitoring environmental and crop conditions (**Figure 7**). Sensors are deployed in tea plantations to measure soil moisture, temperature, humidity, and nutrient levels, enabling farmers to make informed decisions regarding irrigation, fertilization, and pest control (Mao et al., 2023). These sensor networks contribute to efficient water management and help prevent crop stress, thereby improving yield and quality. Furthermore, sensor-based disease detection systems allow early identification of pest infestations and plant diseases, reducing the need for excessive chemical applications and supporting sustainable farming practices (Yu et al., 2025; Rahat et al., 2025; Jiang et al., 2025; Yang et al., 2023).

Recent studies demonstrate a growing consensus that integrating UAV-based remote sensing with ML significantly enhances precision tea management compared to conventional approaches. Multi-source data fusion combining multispectral, thermal, RGB, and LiDAR sensors consistently improves the accuracy and robustness of models for estimating canopy structure and physiological traits such as plant height, leaf area index, and chlorophyll content (Li et al., 2023). Similarly, IoT-integrated UAV systems enable real-time environmental monitoring and yield estimation, supporting scalable and modular farm management solutions (Kariyawasam et al., 2025). Deep learning (DL) approaches, particularly CNNs and YOLO-based models, show strong performance in tea disease detection, achieving high accuracy under complex field conditions and reinforcing the value of image-based AI systems in crop health monitoring (Zhang et al., 2026; Debbarma et al., 2025; Guo et al., 2026). Additionally, UAV-enabled multispectral analysis has proven effective for non-destructive assessment of plant health indicators, including nitrogen status and chlorophyll content (Li et al., 2026; Xie et al., 2025).

Despite this consensus, some contradictions and task-specific differences are evident. While multi-source data generally enhances prediction performance, certain traits such as leaf nitrogen content may be more accurately estimated using single-source multispectral data, indicating that increased data complexity does not always yield better results (Li et al., 2023). Moreover, model performance varies by application: traditional ML models like Support Vector Machines (SVM) and Random Forest (RF) often outperform DL in structured trait estimation, whereas CNN-based architectures dominate in image-based disease classification (Li et al., 2023; Zhang et al., 2026). These findings suggest that model selection should be tailored to the specific agronomic task rather than relying on a single universal approach. Several research gaps remain. Most studies are conducted at small or experimental scales, limiting their applicability across diverse tea-growing environments. There is also a lack of integration between UAV-ML outputs and practical farm-level decision support systems, particularly in resource-constrained settings (Wu et al., 2025; Xiao et al., 2025; Han et al., 2025). **Table 3** presents a summary of some of the key ML and DL models applied to tea leaf disease detection and quality monitoring.

**Table 3:** Tea leaf disease detection & quality monitoring using ML/DL models.

Model/technique	Target task	Dataset	Key finding	Reference
SVM (with SLIC segmentation)	Disease detection	Tea leaf image dataset	Achieved 95.46% classification accuracy	Sun et al., 2019
Optimised Segmentation + ML	Disease detection	Adapted image-based methods for tea	93.97% accuracy with improved feature selection	Sharif et al., 2018
Random Forest (RF)	Quality classification	NIR spectroscopy dataset	High classification accuracy for tea quality assessment	Chen et al., 2021
Hybrid SVM + Filtering	Disease detection	Tea leaf dataset	Improved detection accuracy compared to baseline models	Prabu et al., 2022
CNN (basic architecture)	Disease classification	Tea leaf images	Higher accuracy than traditional ML methods	Latha et al., 2021
GoogLeNet (Inception)	Disease classification	Leaf/plant image datasets	High performance in disease classification tasks	Salka et al., 2025
ResNet/EfficientNet	Disease classification	Leaf disease datasets	Superior performance over conventional CNNs	Sinha and Patil, 2024
Deep Neural Network (DNN)	Disease identification	Tea leaf dataset	High accuracy in automatic disease detection	Datta and Gupta, 2023
CNN + Feature Fusion	Disease classification	Tea leaf dataset	Enhanced classification performance through feature integration	Yücel and Yıldırım, 2023
Federated Learning + CNN	Disease severity analysis	Distributed tea datasets	Enables privacy-preserving and efficient model training	Vats et al., 2024
Lightweight VGG-ICNN	Disease classification	Crop/tea datasets	Efficient and deployable model for real-time use	Thakur et al., 2023
YOLOv3/YOLOv5 (YOLO-Tea)	Real-time detection	Field tea leaf dataset	Fast detection with high precision	Xue et al., 2023
Faster R-CNN (TAME)	Object detection	Tea leaf dataset	High accuracy in detecting small diseased regions	Liu et al., 2024a
Mask R-CNN	Disease segmentation	Leaf disease datasets	Accurate segmentation of infected regions	Sahu and Pandey, 2023
SVM and CNN (ResNet50) with Computer Vision	Classification of oolong tea varieties	Tea image datasets	ResNet50 achieved >93% Top-1 accuracy, enabling rapid and accurate oolong tea variety classification.	Zhu et al., 2024
Improved YOLOv8x-SPPCSPC-CBAM (YOLOv8x enhanced with SPPCSPC and CBAM modules)	Quality grade classification	Tea image	99.1% mAP, 99.1% precision, and 97.7% recall for accurate fresh tea leaf grade classification.	Zhao et al., 2024
CNN + Attention (CAM/SAM/CBAM)	Feature extraction & classification	Image & spectral datasets	Enhances feature representation and accuracy	Zhang et al., 2023a
CNN (quality evaluation)	Quality assessment	Tea image dataset	Accurate classification of external quality attributes	Bhargava et al., 2024
LSTM	Quality prediction	Spectral/sensor datasets	Captures temporal patterns in tea quality	Hu et al., 2023
DBN (Deep Belief Network)	Chemical property prediction	Spectral datasets	Effective regression for chemical composition	Chen et al., 2015
CNN + Spectroscopy (NIRS)	Quality grading	Chemical composition dataset	High accuracy in grading tea quality	Liu et al., 2024a
CNN	Variety classification	Tea image dataset	96.3% accuracy (F1 = 0.95) for nine tea varieties	Dong, 2024
MobileNetV3/EfficientNetV2	Adulteration detection	Tea image dataset	Effective detection of counterfeit black tea and adulteration levels	Besharati et al., 2024

Some ML/DL architectures were originally developed using non-tea or general crop datasets but are included due to their broad applicability and proven effectiveness in plant disease detection, image classification, and precision agriculture tasks relevant to tea research.

Intelligent tea harvesters equipped with AI technologies are being developed to perform selective plucking, targeting only the tender leaves required for high-quality tea. Although these technologies are still evolving, they hold great potential to address labour shortages and improve harvesting efficiency (Abhiram, 2024). Despite these advancements, several challenges remain in the adoption of modern technologies in tea farming. High initial investment costs, limited technical expertise, and limited access to digital infrastructure can hinder the widespread adoption of these innovations, particularly among smallholder farmers (Dechassa and Merga, 2022). Additionally, issues such as data accuracy, weather interference in UAV operations, and the need for standardised datasets for ML models present ongoing challenges (Yang et al., 2023). The future of precision tea agriculture will rely on integrating compact AI models with IoT, UAVs, and multimodal imaging to enable real-time, in-field monitoring and decision-making. These technologies improve the detection of diseases, nutrient deficiencies, and pests while allowing deployment on low-cost edge devices, making them suitable for remote areas. Additionally, combining multiple data types (e.g., RGB, multispectral, thermal) enhances accuracy (Kalita et al., 2025).

Despite significant advances, important knowledge gaps remain, including the lack of large, standardized datasets and limited validation of ML models across diverse tea-growing environments. Conflicting findings indicate that traditional ML methods (e.g., SVM and RF) may outperform deep learning models for some prediction tasks, while CNN- and YOLO-based approaches are more effective for image-based disease detection, highlighting the need for application-specific model selection.

## 11 Pest and disease management

Pest and disease management is a critical component of tea cultivation, as tea plants are highly susceptible to a wide range of insects, fungi, and other pathogens that can significantly reduce yield and quality.

### 11.1 Modern pest and disease control methods

With the intensification of tea farming and increasing demand for higher productivity, modern pest and disease control methods have been widely adopted. These methods primarily involve the use of chemical pesticides and fungicides, which provide rapid and effective control of pests and diseases. Modern techniques also include the use of improved spraying equipment, monitoring tools, and scientific recommendations for pest control (Deka et al., 2021; Chen and Luo, 2025; Ahale et al., 2024). However, excessive reliance on chemical inputs has led to several issues, including pesticide resistance, environmental pollution, contamination of soil and water, and concerns about food safety and human health. Additionally, the cost of chemical inputs can place a financial burden on farmers, particularly smallholders.

### 11.2 Integrated pest management

To address the limitations of both traditional and purely chemical approaches, Integrated Pest Management (IPM) has emerged as a sustainable and balanced strategy in tea farming. IPM combines biological, cultural, mechanical, and chemical methods to manage pests in an economically viable and environmentally sound manner (Mamun et al., 2014; Al Mamun et al., 2025). The core principle of IPM is to minimise pesticide use by applying chemicals only when pest populations exceed economic threshold levels. This approach involves regular monitoring of tea plantations, early detection of pest and disease symptoms, and the use of biological control agents such as natural predators, parasitoids, and microbial pesticides (Babu, 2021; Chen and Luo, 2025; Deguine et al., 2021). Cultural practices, including proper pruning, sanitation, and soil management, are also integrated into IPM to reduce pest habitats and improve plant health. IPM not only reduces the environmental impact of pest control but also helps maintain ecological balance and long-term soil fertility (Chen and Luo, 2025). By optimizing input use and reducing dependency on chemicals, IPM enhances sustainability while maintaining productivity. However, successful implementation of IPM requires technical knowledge, farmer training, and access to reliable monitoring systems, which can be challenging in some regions (Guastella et al., 2017).

## 12 Challenges associated with tea farming

Tea farming faces numerous interconnected challenges that threaten its sustainability, productivity, and economic viability. One of the most critical issues is climate change, which significantly affects tea cultivation due to the crop’s sensitivity to environmental conditions. Variations in temperature, irregular rainfall patterns, and increased frequency of extreme weather events have led to fluctuations in both yield and quality (Ashardiono and Cassim, 2014; Ahmed et al., 2018). Changes in climatic conditions also influence the biochemical composition of tea leaves, affecting flavour and market value. Additionally, climate change contributes to the emergence and spread of pests and diseases, further increasing production risks and costs.

### 12.1 Labour

Tea farming faces a range of interconnected challenges that affect productivity, profitability, and sustainability across major tea-producing regions. One of the most significant issues is labour shortage, as tea cultivation, particularly harvesting, remains highly labour-intensive and requires skilled workers to selectively pluck tender leaves (Zhang, 2025; Tatoglu, 2025). Younger generations are increasingly moving away from agricultural jobs toward urban employment, leading to a decline in available labour and rising wage costs, which significantly increase production expenses. In fact, harvesting alone can account for a substantial portion of total production costs, making labour scarcity a critical constraint in the tea industry (Ganewatta and Edwards, 2000).

Labour remains the dominant cost component in tea production. The Indian Tea Association reports that Labour-related expenditures account for nearly 60% of total tea production costs in India’s organised plantation sector, largely due to statutory obligations covering wages, housing, healthcare, sanitation, and welfare provisions under the Plantation Labour Act. The report further notes that harvesting and field Labour shortages have intensified production costs, with wage expenditures in Indian Assam and West Bengal increasing by more than 25% between 2018 and 2019 alone (Indian Tea Association, 2019).

### 12.2 Climate change

**Figure 8:**
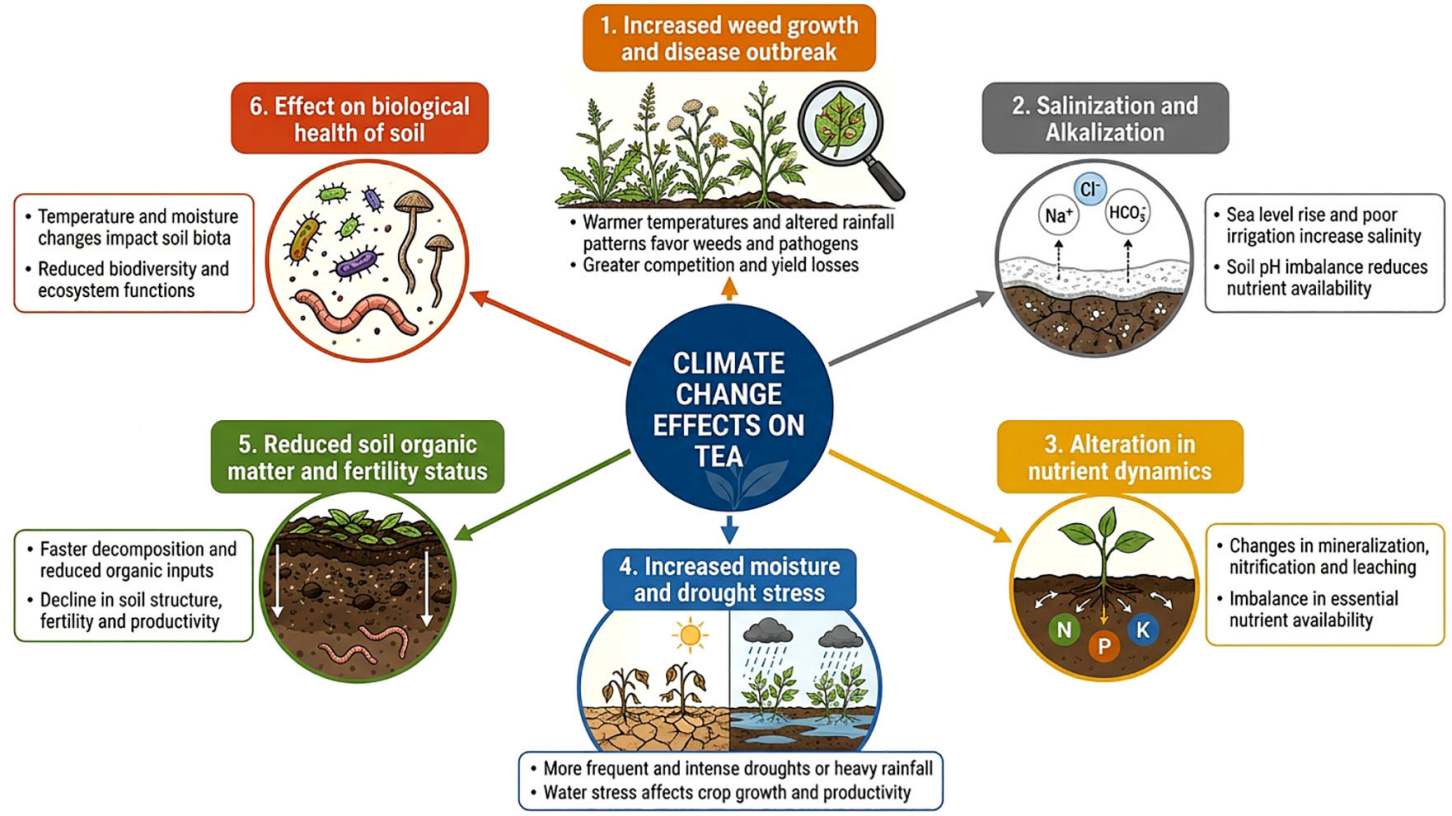
Impacts of climate change on agriculture, including increased weed growth and disease outbreaks, altered nutrient dynamics, soil salinization and alkalization, reduced soil organic matter and fertility, moisture imbalance, and heightened drought stress.

Climate change is one of the most critical and complex challenges facing tea farming today because tea plants are highly sensitive to environmental conditions such as temperature, rainfall, humidity, and altitude. Even small changes in these factors can significantly affect both the yield and quality of tea. One of the primary impacts of climate change is rising temperatures, which alter the growth cycle of tea plants (Han et al., 2018a; Ahmed et al., 2018). While moderate warmth can enhance growth, excessive heat accelerates leaf maturation, leading to coarser leaves with lower concentrations of desirable compounds such as catechins and amino acids. This directly reduces tea quality, affecting flavour, aroma, and market value. In addition, higher temperatures can shift suitable tea-growing regions to higher altitudes, potentially rendering some traditional tea-producing areas less productive over time. Another major issue is the increasing irregularity of rainfall patterns (Ashardiono and Cassim, 2014; Rajan et al., 2024). Tea cultivation depends on consistent and well-distributed rainfall throughout the year. However, climate change has led to more frequent droughts and unpredictable rainfall, which disrupt plant growth and reduce yields (**Figure 8**). Prolonged dry periods cause water stress in tea bushes, limiting leaf development and lowering productivity (Jayasinghe and Kumar, 2020; Ahmed et al., 2018). On the other hand, excessive rainfall can lead to waterlogging, soil erosion, and nutrient leaching, all of which damage root systems and reduce soil fertility. In hilly tea-growing regions, heavy rainfall can also trigger landslides, further threatening plantations and infrastructure. These fluctuations in water availability make it difficult for farmers to maintain stable production cycles. Additionally, pest and disease pressures pose a continuous threat to tea plantations. Tea crops are vulnerable to a wide range of pests and diseases, which can cause significant yield losses if not properly managed. In many cases, farmers rely on chemical pesticides (Dechassa and Merga, 2022; Ahmed et al., 2018).

Environmental degradation also poses significant challenges. Tea is commonly grown as a monoculture crop, which can lead to soil fertility loss, increased erosion, and reduced biodiversity. The extensive use of fertilizers and pesticides contributes to soil and water pollution, negatively affecting ecosystems and human health. Furthermore, deforestation for plantation expansion and fuelwood use in processing exacerbates ecological imbalance (UNCTAD, 2016). Climate change is expected to significantly reduce tea productivity across major producing regions, with FAO projections indicating declining climatic suitability in Assam and Kenya by 2050 and yield reductions of approximately 10–22% in Sri Lanka’s low- and mid-elevation tea-growing areas due to rising temperatures and moisture stress. Historical trends already show rainfall declines exceeding 200 mm and minimum temperature increases of about 1.3 °C in Northeast India, intensifying drought stress, pest outbreaks, and production instability (FAO, 2016).

### 12.3 Economic and technological challenges

Economic and market-related challenges further impact tea farming. Producers face fluctuating global tea prices, changing consumer preferences, and increasing competition among producing countries (Que and Zhao, 2024). Maintaining productivity while remaining competitive in the global market is a persistent challenge. Rising input costs, including labour and fertilizers, along with regulatory changes such as restrictions on agrochemicals, also affect profitability and production efficiency (Chang, 2015). Finally, technological limitations and knowledge gaps hinder the modernization of tea farming. Many tea-growing regions still rely on traditional cultivation and monitoring methods, limiting efficiency and scalability. The adoption of advanced technologies such as ML, remote sensing, and automated disease detection systems remains limited, despite their potential to improve productivity and sustainability (Kalita et al., 2025).

## 13 Conclusion

This review identifies several critical insights that shape the future trajectory of tea production systems. First, tea cultivation is fundamentally shaped by the interaction between environmental conditions, plant physiology, and management practices, indicating that site-specific and adaptive agronomy is essential for sustaining both yield and quality. Second, the comparison between conventional and organic systems reveals that productivity sustainability trade-offs are context-dependent, and optimal outcomes are achieved not by strict adherence to one system but through integrated, resource-efficient approaches. Third, emerging technologies, particularly AI, remote sensing, and precision agriculture, are transforming tea production by enabling data-driven decision-making, real-time monitoring, and improved resource use efficiency, although their adoption remains uneven. Fourth, persistent constraints such as climate variability, labour limitations, and technological accessibility highlight the need for scalable and inclusive innovations tailored to diverse production systems. Collectively, these insights suggest that the long-term resilience of the tea sector will depend on integrating traditional knowledge with advanced technologies, fostering a multidisciplinary framework that supports sustainable intensification and adaptive management.

## 14 Future perspectives

Future research in tea production should prioritise the development of climate-resilient cultivation systems that can withstand increasing variability in temperature, rainfall, and extreme weather events. This includes breeding and selecting stress-tolerant tea cultivars, improving water-use efficiency through precision irrigation, and optimizing shade and soil management practices. A second priority is the advancement of integrated digital agriculture, where AI, remote sensing, and IoT technologies are combined into scalable decision-support systems. Research should focus on improving model transferability across regions, reducing data dependency, and designing cost-effective tools suitable for smallholder farmers.

Future research should focus on developing robust multimodal frameworks that integrate UAV, IoT, sensor, and imaging data, while evaluating their performance across multiple tea-growing regions and seasons. Greater attention should also be given to explainable AI, edge-computing deployment, federated learning, and decision-support systems that translate model outputs into actionable recommendations for farmers. Such efforts would enhance the scalability, reliability, and practical adoption of precision agriculture technologies in the tea sector.

Another key area is the refinement of sustainable intensification strategies, particularly the integration of organic and conventional practices to balance productivity with environmental conservation. Long-term, multi-location studies are needed to quantify trade-offs in yield, soil health, biodiversity, and economic returns under different management systems. Finally, there is a need for standardised datasets and open data platforms to support reproducibility, cross-regional comparisons, and collaborative innovation. Collectively, these priorities highlight the importance of a multidisciplinary approach that integrates agronomy, data science, engineering, and socioeconomics to ensure the long-term sustainability and resilience of global tea production systems.
